# Prevalence of depressive symptoms and their associated factors among older adults in Yirgalem town, Southern Ethiopia: A community-based cross-sectional study

**DOI:** 10.3389/fpsyt.2023.1148881

**Published:** 2023-03-29

**Authors:** Abiy Mulugeta, Telake Azale, Yohannes Mirkena, Selam Koye, Girum Nakie, Abenet Kassaye, Jerman Dereje, Neim Bedewi, Deribe Bekele Dechasa, Henock Asfaw

**Affiliations:** ^1^Department of Psychiatry, School of Nursing and Midwifery, College of Health and Medical Science, Haramaya University, Harar, Ethiopia; ^2^Department of Health Education and Behavioural Sciences, College of Medicine and Health Science, University of Gondar, Gondar, Ethiopia; ^3^Department of Psychiatry, College of Medicine and Health Science, University of Gondar, Gondar, Ethiopia; ^4^Department of Psychiatric Nursing, College of Medicine and Health Science, Wollo University, Dessie, Ethiopia; ^5^Department of Clinical Pharmacy, School of Pharmacy, College of Health and Medical Science, Haramaya University, Harar, Ethiopia; ^6^School of Nursing and Midwifery, College of Health and Medical Science, Haramaya University, Harar, Ethiopia

**Keywords:** older adults, prevalence, associated factors, Yirgalem, depressive symptoms

## Abstract

**Background:**

Depression is a serious mental health issue and the largest contributor to disability worldwide. Elderly people with depression are significantly more likely to experience negative outcomes such as poor physical health, strained social relationships, and decreased quality of life. Studies on geriatric depression are limited in developing nations like Ethiopia.

**Objectives:**

The purpose of this study was to determine the prevalence of depressive symptoms and associated factors among older adults in Yirgalem, Southern Ethiopia, in 2022.

**Methods:**

A community-based cross-sectional study was conducted on a sample of 628 older adults in Yirgalem town from May 15 to June 15, 2022. The study subjects were selected using a multi-stage systematic sampling technique. Data were collected using the 15-item Geriatric depression scale through face-to-face interviews. The collected data were edited, cleaned, coded, and entered into Epi data version 4.6 software and analyzed using STATA version 14. Bivariable and multivariate logistic regression analysis was computed to identify factors associated with depression, and statistical significance was declared at a 95% confidence interval with a *P*-value less than 0.05.

**Results:**

A total of 620 older adults were included in the study, with a response rate of 97.8%. The prevalence of depressive symptoms among older adults was 51.77% (95% CI: 47.83–55.69). Being a woman (AOR = 2.3, 95% CI: 1.56–3141); being of more advanced age: 70–79 years old (AOR = 1.92, 95% CI: 1.20–3.07), 80–89 years old (AOR = 2.15, 95% CI: 1.27–3.65), 90 and older (AOR = 3.77, 95% CI: 1.95–7.79); living alone (AOR = 1.99, 95% CI = 1.17–3.41); having a chronic illness (AOR = 3.24, 95% CI: 1.06–4.46); having anxiety (AOR = 3.40; 95% CI: 2.25–5.14); and having poor social support (AOR = 3.56, 95% CI: 2.09–6.04) were statistically associated with depressive symptoms at a *P*-value of less than 0.05.

**Conclusion:**

This study found that depression affects more than half of the elderly residents in the study area. More advanced age, being a woman, living alone, having a chronic illness, having anxiety, and having poor social support were all strongly linked to depression. There is a need to integrate counseling and psychiatric services into the community healthcare system.

## Introduction

Aging is a universal, progressive, and detrimental process that occurs throughout one’s life. It is a naturally occurring phenomenon, and getting older is the stage in life when most aspects of life require the assistance of others (1). High rates of population growth in most industrialized and developing nations are a major focus of health services, particularly mental health care services. An estimated 12.3% of the world was at or above the age of 60 in 2015, and by 2050, 8 in 10 older adults worldwide are estimated to reside in developing areas (2). In Ethiopia, old age starts at the retirement age, which is 60 (3). Older adults, as opposed to their younger counterparts, commonly face multifarious challenges that are associated with psychological and physical changes. Socio-economic status changes, physical health problems, and the inability to work because of progressive disability are commonly linked with the aging process, which can interpose with the emotional stability of the elderly and have an outcome of overt depression (4).

Depression is manifested primarily by a depressed mood or a lack of interest in previously pleasurable activities. Furthermore, depressed people may have reduced energy; difficulties with concentration, sleep, appetite, and weight changes; feelings of regret and dejection; and suicidal ideation (5). Depression is caused by a complex interaction of biological genetic susceptibility and life situations or a person’s social and internal world, which is a key cause of depressive risk throughout the lifespan (6, 7). Late-life depression is distinguished by symptoms such as sleep disturbance, decreased appetite, lack of energy, somatic complaints, pessimism about the future, and impairment in cognitive functions such as memory (8, 9).

Globally, depression is a serious mental health issue, the third leading cause of disease burden, and the largest contributor to disability (10). As per the estimation of the World Health Organization (WHO), the global magnitude of depression among older individuals ranges between 10 and 20% depending on sociocultural settings, and the Disability Adjusted Life Years (DALYs) of depression for older people is approximately 9.17 million years, or 1.6% of the total DALYs in this age group (11).

A review of 42 studies revealed that the prevalence rate of depressive disorders among the elderly population is approximately 31.7% (12). A meta-analysis study conducted in African countries showed that the pooled prevalence of depression among older adults is about 26.3% (13). According to studies conducted in Ethiopia, the prevalence of depression among the elderly population ranges between 28.5 and 54.6% (14, 15).

There are several risk factors affecting depression in older age groups, which vary contextually. The risk of having depression is higher among the elderly who are women; are of advanced age; never married; are widowed, divorced, or separated; have a low education status; and are unemployed and low-income (16–18). Studies conducted in developing countries have shown that physical restriction in instrumental activities, other medical conditions like having one or more chronic conditions, substance use, multiple hospitalizations, and disability are some of the important determinants of old-age depression. Death of a spouse and being financially dependent on others are commonly experienced serious life events for older people who are diagnosed with depression (19–21). Studies conducted during the COVID-19 pandemic identified general health status, previous history of depression, self-harm and suicidal attempts, family responsibility, economic change, age, and lower scores for perceived control over one’s health and social life as strong determinants of depressive symptoms among older people (22, 23).

The complications of depression are more severe among elderly people than among their middle-aged counterparts. Depression significantly increases the risk of negative outcomes in older adults, such as cognitive deficits, disrupted social functioning, and disability, resulting in a greater burden on family and caregivers (24, 25).

Elderly people suffering with depression have reduced life satisfaction and quality, social deprivation, increased utilization of health services and associated costs, and limitations to activities of daily living (ADL). Furthermore, it can negatively affect the prognosis and recovery of other chronic illnesses by amplifying the perception of poor health and interfering with their adherence to medications (26).

Consequently, depression in older people significantly increases mortality related to suicidal and non-suicidal causes compared to their younger counterparts (27, 28). Although older adults living in both community and institutional settings are more likely to experience depression, most of them have been unrecognized and untreated since there is a dearth of adequate and evidence-based information (29). While there have been many studies conducted in industrialized countries, the burden of depression among the elderly, particularly in LMICs, including Ethiopia, is not well-addressed. Hence, this study aimed to assess the prevalence of depressive symptoms and their associated factors among older adults in Yirgalem town from May 15 to June 15, 2022.

## Materials and methods

### Study area and period

The research was carried out in Yirgalem town, which is one of the oldest settlements in Sidama National Regional State, Southern Ethiopia. Geographically, the town is situated approximately 325 kilometers from Addis Ababa, the nation’s capital, and 47 km from Hawassa City, the state seat of Sidama National Regional State. According to the 2019 Yirgalem town health office report, the entire population of the town is predicted to be 79,605, with 39,166 men and 40,439 women. The data from local health extension workers showed that there were 3,236 older adults in the town. The town contains 13 kebeles (Ethiopia’s smallest administrative unit), one governmental general hospital with a psychiatric clinic, and one health center. The study was conducted from May 15 to June 15, 2022.

### Study design and population

A community-based cross-sectional study design was employed. All elderly people (aged 60 and above) who resided in Yirgalem town, Southern Ethiopia were considered the source population. The study participants were elderly people who had been living in Yirgalem town, Southern Ethiopia during the 6 months before the data collection period and who were available during the data collection period. Older adults who were unable to communicate and seriously ill when the data were collected were excluded from the study.

### Sample size determination

When determining the sample size for this study, the outcome variable and the variables that were significantly associated with the outcome variable were taken into consideration. The sample sizes for the first and second objectives were calculated separately. A single population proportion was employed to calculate the sample size for the first specific objective of the study. In this study, we used the proportion of depression from the study conducted in North Shoa, which was 54.6% (15).

Therefore,


n=( 1.96)2⁢ 0.546⁢(1-0.546)0.052=381

Since we used a multistage sampling technique by taking a design effect of 1.5, and adding a 10% non-response rate, the calculated final sample size was 628.

The sample size for the second specific objective was determined using the Epi Info version 7, considering the factors that were significantly associated with depression at (*p* = 0.05). Therefore, this study used the single population formula of the sample size since 628 is greater than the sample size calculated by the double population formula.

### Sampling procedure

A multi-stage systematic random sampling technique was used to collect the required sample size. First, 5 kebeles were selected randomly using a lottery method from the total 13 kebeles of Yirgalem town. Then, the sample size was allocated to the randomly selected kebeles based on their elderly population proportion, and a systematic random sampling technique was used to select the individuals. The sampling interval (k) was determined by the study population (1,459 old age individuals in the selected Kebeles) divided by sample size (1459/628) = 2.3). Then, the data were collected from every two older adults. The lottery method (simple random sampling technique) was used to select the first elderly participant, and one eligible individual was selected from each household using the lottery method for the case where more than one older adult was living in the house.

### Data collection tools and procedure

The Data were collected using a structured questionnaire in Amharic. The questionnaire has five sections: the first contains socio-demographic characteristics; the second contains the dependent variable depression and was measured by the Geriatric Depression Scale (GDS-15) in the form of dichotomous variable (yes/no).

The geriatric depression scale has 15 items, five of which are negatively worded questions. Even though GDS is not validated in this country, several studies from Asian and many African countries with diverse sociocultural backgrounds were employed to screen depression. It has been tested and validated among older Asian adults, with sensitivity of 97%, specificity of 95%, and a Cronbach alpha value of 0.80 (30). In this study, a pre-test was carried out to check the suitability of GDS with a Cronbach-alpha value of 0.81.

Anxiety was assessed using the GAD-7 tool, a seven-item questionnaire to measure the level of anxiety in the respondents during the preceding 2 weeks. Each item is scored from 0 (not at all) to 4 (nearly every day) and scores of all items are added to get the total scores, ranging from 0 to 21, with a cut point of 10 and above (31). The tool has a sensitivity value of 0.89 and a specificity value of 0.82 for identifying GAD (32). Several studies in Ethiopia have used GAD-7 to assess anxiety symptoms in different populations.

Stressful life events were assessed using the List of life-threatening experiences (LTE). The LTE includes 12 significant life events that occurred over the previous 6 months, which are rated by yes or no responses, with a cut point of one. The LTE has high test-retest reliability (Kappa: 0.61–0.87) as well as predictive validity, and numerous Ethiopian studies have employed LTE to measure adverse life events in various populations (33). The last section contains items assessing substance use. Data were collected *via* face-to-face interviews, using the Amharic version of the structured questionnaire, by six BSc nurses. Data collectors and the supervisor were trained on the purpose of the study, consent forms, maintaining confidentiality, and the overall data collection procedures.

### Data quality control

The questionnaire was initially prepared in English and translated into the Amharic language for the participants by two language experts. The pre-test was conducted on 5% of the study sample in Wendo Genet town 1 week before the data collection. Training was delivered to data collectors and supervisors by the principal investigator about how to use questionnaires, the ethical principle of confidentiality, and data management prior to data collection. The data collectors were supervised daily, and the filled questionnaires were checked daily by the supervisors and the principal investigator for completeness.

### Data processing and analysis

Data were checked, coded, and entered into Epi-Data version 4.6 and exported to Stata version 14 for analysis. The socio-demographic characteristics and other factors of respondents were analyzed by descriptive statistics.

Bi-variable logistic regression analysis was performed to identify the association of each independent variable with the outcome variable. All variables with a *p*-value of less than 0.2 at bivariable logistic regression analysis were entered into the multivariable logistic regression model. A *p*-value of <0.05 was considered statistically significant, and the adjusted odds ratio (AOR), with a 95% confidence interval (CI), was calculated. The goodness of fit model was checked using the Hosmer-lemshow test.

### Operational definition

Depression: A score of five and above on the Geriatric Depression Scale (GDS-15) (5).

Anxiety: Participants who scored 10 and above on the Generalized Anxiety Disorder Scale (GAD-7) were considered as having anxiety.

Social support: The Oslo-3 social support scale was used to score social support on a scale ranging from 3 to 14 with three categories: scores of 3–8 were considered poor support, 9–11 moderate support, and 12–14 strong support (34).

Stressful life event: Using the List of Threatening Experiences (LTE), if the respondent had experienced one or more major life events within the previous 6 months from a total of twelve items, they were considered as having experienced a serious life event (35).

Previous use of substances: Having used at least one specific substance for a non-medical purpose at least once in a lifetime (alcohol, khat, tobacco, others) (36).

Current Substance use: Having used at least one specific substance for non-medical purposes within the last 3 months (alcohol, khat, tobacco, others) (36).

## Results

### Socio-demographic characteristics of the participants

From a total of 628 samples, 620 participants were included in the study, with a response rate of 98.7%. There were more men, at 52.25 % (324). The mean age and standard deviation of the study participants were found to be 72.8 and ±9.83 years, respectively, with ages ranging from 60 to 96 years. Regarding the living arrangements of participants, 83.71% (519) were living with family (spouse and children). Regarding religion, 298 participants (48.06%) were orthodox and 37.9% (235) were protestant. A total of 336 (54.19%) participants were married. Over half, 56.61% (351), of participants had had formal education and 40.65% (252) respondents were financially supported by their family, as shown in Table 1.

**TABLE 1:** Socio-demographic characteristics of older adults in Yirgalem town, Southern Ethiopia 2022 (*n* = 620).

Variable	Category	Frequency (*n*)	Percentage (%)
Gender	Men	324	52.25
	Women	296	47.75
Age	60–69	226	36.45
	70–79	195	31.45
	80–89	129	20.81
	≥90	70	11.29
Marital status	Single	64	10.32
	Married	336	54.19
	Divorced	86	13.87
	Widowed	134	21.62
Living arrangement	With family	519	83.71
	Alone	101	16.29
Religion	Orthodox	298	48.06
	Protestant	235	37.90
	Muslim	63	10.16
	Other	24	3.88
Source of income	Retirement	180	29.03
	Helped by family	252	40.65
	Private business/self-work	188	30.32
Educational status	No formal education	269	43.39
	Literate/formal education	351	56.61

Others: Catholic, Adventist, Jehovah’s witness.

### Psychosocial and substance use characteristics of respondents

Regarding social support, (259) 41.77% had moderate social support and (196) 31.61% had poor social support. Regarding serious life events, (243) 39.2% of respondents had experienced serious life events in the previous 6 months. Regarding participant substance use, 45.48% (282) reported ever having used alcohol and 34.68% (215) were current alcohol users, as shown below in Table 2.

**TABLE 2:** Psychosocial and substance use characteristics of older adults in Yirgalem town, Southern Ethiopia 2022 (*n* = 620).

Explanatory variables	Category	Frequency (n)	Percentage (%)
Social support	Poor	196	31.61
	Moderate	259	41.77
	High	165	26.62
Stressful life event	Yes	243	39.2
	No	377	60.8
Lifetime alcohol use	Yes	282	45.48
	No	338	54.52
Lifetime tobacco use	Yes	52	91.59
	No	566	8.41
Lifetime khat use	Yes	92	14.84
	No	528	85.16
Current alcohol use	Yes	215	34.68
	No	405	65.32
Current tobacco use	Yes	37	5.98
	No	582	94.02
Current khat use	Yes	71	11.45
	No	549	88.55

### Clinical factors of respondents

The majority, 609 (98.2%), of the respondents had no known history of mental illness, whereas 241 (38.87%) had a history of known chronic disease. Of the total study participants, 128 (20.64%) and 61 (9.83%) had suicidal ideation and attempts, respectively. More than one-third, 218 (35.16%), of the respondents were found to have anxiety. The majority, 603 (97.26%), of subjects had no family history of mental illness (Table 3).

**TABLE 3:** Clinical factors of older adults in Yirgalem town, Southern Ethiopia 2022 (*n* = 620).

Variable	Category	Frequency (n)	Percentage (%)
History of mental illness	Yes	11	1.77
	No	609	98.23
History of known chronic disease	Yes	241	38.87
	No	379	61.13
Suicidal ideation	Yes	128	20.64
	No	494	79.36
Suicidal attempt	Yes	61	9.83
	No	559	90.17
Anxiety	Yes	218	35.16
	No	402	64.84
Family history of mental illness	Yes	17	2.74
	No	603	97.26

### Prevalence of depression

The findings of this study revealed that the prevalence of depressive symptoms among older adults was 51.77% (95% CI: 47.83–55.69%).

### Factors associated with depression

In bivariate logistic regression analysis, variables such as age, being a woman, being divorced, being single, being widowed, source of income, living alone, ever having and recently having used alcohol, having known chronic disease, having attempted suicide, having anxiety, having and poor social support had a *P*-value of less than 0.2. These variables fulfill the minimum requirements for further multivariate logistic analysis. Among these variables, advanced age, being a woman, living alone, having known chronic disease, having anxiety, and having poor social support were statistically significant for depressive symptoms in multivariable analysis, with a *p*-value less than 0.05.

In this study, the odds of having depressive symptoms among women were approximately 2.31 times higher compared to men (AOR = 2.31, 95% CI: 1.56–3.41). As age advanced, the odds of having depressive symptoms increased, and elderly individuals who were 70–79 years of age were 1.92 times (AOR = 1.92, 95% CI: 1.20–3.07) more likely to develop depression than those 60–69 years old, whereas those aged 80–89 years were 2.15 times (AOR = 2.15, 95% CI: 1.27–3.65) more likely, and those aged 90 and older were 3.77 times (AOR: 3.77, 95% CI: 1.95–7.29) more likely. In this study, the odds of having depression among elderly participants who lived alone were two times higher than among participants living with family (AOR = 1.99, 95% CI: 1.17–3.41).

The result of this study revealed that elderly respondents who had known chronic disease were 3.2 times more likely to develop depression compared to those who had no known chronic disease (AOR = 3.24, 95% CI: 1.06–4.46). The odds of having depression among elderly respondents who had anxiety were 3.4 times higher than those who had no anxiety (AOR = 3.40, 95% CI: 2.25–5.14). Social support was another factor associated with elderly depression. Participants with poor social support were 3.56 times more likely to have depression compared to participants with strong social support (AOR = 3.56, 95% CI: 2.09–6.04), as shown in Table 4.

**TABLE 4:** Bivariate and multivariate logistic regression analysis of factors associated with depression among older adults in Yirgalem town Southern Ethiopia, 2022 (*n* = 620).

Explanatory variables	Category	Depression		COR (95% CI)	AOR (95% CI)
		**Yes**	**No**		
Gender	Men	130	194	1	1
	Women	191	105	2.71 (1.96–3.75)	**2.31 (1.56**–3.41)**
Age	60–69	84	142	1	1
	70–79	112	83	2.28 (1.54–3.37)	**1.92 (1.20**–3.07)**
	80–89	76	53	2.42 (1.55–3.77)	**2.15 (1.27**–3.65)**
	≥ 90	49	21	3.94 (2.21–7.03)	**3.77 (1.95**–7.29)**
Marital status	Married	149	187	1	1
	Single	43	21	2.56 (1.46–4.51)	1.75 (0.90–3.42)
	Divorced	50	36	1.74 (1.07–2.81)	0.70 (0.38–1.28)
	Widowed	79	55	1.80 (1.20–2.70)	1.10 (0.66–1.87)
Living arrangement	Living with family	254	265	1	1
	Living alone	67	34	2.05 (1.31–3.21)	**1.99 (1.17**–3.41)*
Source of income	Retirement	90	90	1.04 (0.69–1.57)	1.09 (0.66–1.8)
	Helped by family	139	113	1.28 (0.87–1.87)	1.26 (0.79–2.02)
	Private business/self-work	92	96	1	1
Chronic disease	Yes	172	69	3.84 (2.70–5.42)	**3.24 (2.15**–4.88)**
	No	149	230	1	1
Suicidal attempt	Yes	38	23	1.61 (1.04–2.98)	1.23 (0.63–2.37)
	No	283	276	1	1
Anxiety	Yes	157	61	3.73 (2.61–5.33)	**3.40 (2.25**–5.14)**
	No	164	238	1	1
Social support	Strong	58	107	1	1
	Moderate	120	139	1.59 (1.06–2.38)	1.49 (0.93–2.39)
	Poor	143	53	4.97 (3.17–7.79)	**3.56 (2.09**–6.04)**
Ever use of alcohol	Yes	157	125	1.33 (1.01–2.34)*	1.00 (0.67–1.55)
	No	164	174	1	1
Recent use of alcohol	Yes	124	91	1.43 (1.03–2.00)*	1.25 (0.79–1.98)
	No	197	208	1	1

*p > 0.01, and **p < 0.001, Hosmer-lemshow test = 0.82.

## Discussion

The findings of this study revealed that the prevalence of depression among elderly people was 51.77%, with a 95% CI: 47.83–55.69. We found that being a woman, being of advanced age, living alone, having a chronic disease, having anxiety, and having poor social support were the associated factors of depression in this study. Regarding the prevalence of depression, the finding of the current study is in line with studies conducted in Nepal, 53.1% (37); South Africa, 51.9% (38); and North Shoa, 54.6% (15). The finding of this study is higher when compared to studies conducted in Harar, 28.5% (13); Ambo, 41.8% (39); Dega Damot, 45.9% (13); Wenberema, 45% (17); Ghana, 37.8% (40); Egypt, 44.4% (16); and Morocco, 39.9% (41). Possible reasons for the discrepancies in these results might be differences in socio-demographic characteristics, sample sizes, and measurement scales used.

In the Dega Damot and Wenberema study, a possible reason for the discrepancy might be that the study setting was a rural residence, as well as the differences in sampling techniques used and sample sizes. In the Ghana study, the discrepancy might be attributable to the sample size used, which was 262, and sociocultural differences. This study is also higher than community-based studies conducted in Nigeria, 44.7% (42); Nepal, 41.8% (43); and Singapore, 17.1% (21). The variations seen might be due to differences in measurement scales, sample sizes, sampling techniques, and time variation compared to the current study. In the Nigeria study, the majority of the study participants were aged 60–70 years, and those aged 60–70 were found to be less likely to have depression than those of more advanced age. However, the result of this study was lower than community-based studies conducted in urban India, 75.5% (44); urban Vietnam, 66.9% (20); and Egypt, 62.7% (45) and an institution-based study in Nepal, 74.6% (43). Possible reasons for the discrepancy might be study population, assessment tool variation, and sociocultural and economic differences. In the urban Indian study, the depression assessment tool was GDS-30. In the urban Vietnam study, depression was measured using the Zung Self-depression scale–20 items (ZSDS-20).

On the other hand, the findings of this study were higher than studies conducted in Thailand, 28.5% (46); rural Vietnam, 26.1% (18); India, 42.7% (47); and Sri Lanka, 13.9%. These discrepancies might be due to differences in study sample sizes and screening tools or variations in sociocultural and economic status. In the Sri Lanka study, study participants were between 60 and 74 years old, whereas our study used elderly participants aged 60 and above as the inclusion criteria.

Our study was also higher than studies carried out in Malaysia 27.3% (48), Italy 25.1% (49), China 11.6% (50), and China 46.67% (51). The possible reason for this discrepancy might be variations in study setup, sample sizes, assessment tools, and sociocultural and economical status. In the Malaysia study, the assessment tool for screening depression was the Depression, Anxiety, and Stress scale–21 items (DASS-21).

In the present study, a multivariate logistic regression model revealed that being a woman, being of advanced age, living alone, having a chronic illness, having anxiety, and having poor social support were statistically significant factors associated with depression among the elderly. In this study, women were approximately 2.31 times more likely to have depression than men; this finding was supported by studies conducted in Egypt (16), India (44), Brazil (52), and Ambo (39). A possible justification for this association might be that elderly women had lower education and income levels than elderly men, and due to differences in life expectancy, they seem to be more likely to lose their partners, which is a significant risk factor for depression.

The current study showed that, as age advanced, the odds of having depression increased, and those aged 70–79 years were 1.92 times (AOR = 1.92, 95% CI: 1.20–3.07) more likely to develop depression than those aged 60–69 years old, whereas those aged 80–89 years were 2.15 times (AOR = 2.15, 95% CI: 1.27–3.65) more likely, and those aged 90 and older were 3.77 times (AOR: 3.77, 95% CI: 1.95–7.29) more likely. A possible justification for this finding might be that as age advances, people tend to experience reduced daily functioning abilities, a decreased sense of self-worth, a lower degree of life satisfaction, pain, a lower economic status, and familial losses, which can increase the risk of depression. This was in line with studies conducted in Nigeria (42), Vietnam (20), and India (44). This study also found that the odds of having depression among elderly participants who lived alone were two times higher than among participants living with family (AOR = 1.99, 95% CI: 1.17–3.41). A possible explanation for this link could be that loneliness-related negative emotions have a consistent, recurring structure that is key to the emergence and maintenance of psychopathology. This is in agreement with studies carried out in Ghana (40), Harar (14), and North Shoa (15).

In addition to this, participants with poor social support were 3.56 times more likely to have depression compared to those with strong social support (AOR = 3.56, 95% CI: 2.09–6.04). This might be due to the fact that elderly people have fewer social connections as a result of illness and disability, which leads them to lose intimate ties and their role in society. This could raise the likelihood of elderly people experiencing depression. This is also supported by studies conducted in Morocco (41) and Wemberema (17).

In this study, elderly respondents who had known chronic disease were 3.2 times more likely to develop depression compared to those who had no known chronic disease (AOR = 3.24, 95% CI: 1.06–4.46). This may be explained by the fact that emotional instability or depression may be more likely to develop in those who are physically ill than in those who are relatively healthy. A chronic illness is identified as one of the risk factors for depression development in a WHO report. This is in line with studies carried out in Brazil (52), Sri Lanka (53), and Malaysia (48). Our study also revealed that elderly respondents who had anxiety were 3.4 times more likely to develop depression than those who had no anxiety (AOR = 3.40, 95% CI: 2.25–5.14). This might be due to the fact that elderly people who experience intense worry and anxiety symptoms are more likely to have disabilities, more physical complaints, and lower self-esteem.

### Implication for practice

The findings of our study quantify the burden of depressive symptoms for the benefit of readers, mental health institutions, and policymakers. Furthermore, the results of this study provide information regarding important risk factors for health professionals to design appropriate solutions for the problem, which can improve the mental and physical wellbeing of the elderly population.

## Conclusion

In the present study, depression was shown to affect more than half of the elderly. Advanced age, being a female, living alone, having a chronic illness, having anxiety, and having poor social support were all strongly associated with depression in the elderly. These results emphasize the need to integrate community healthcare services into counseling and psychiatric services. A thorough screening technique to ensure the early identification of depression among the elderly with medical comorbidity is critical, as it contributes to reducing the illness burden on patients’ families and society as a whole.

### Strengths and limitations of the study

This study offers valuable information for mental health institutions and policymakers interested in enhancing the health and quality of life of the elderly population. We recommend that future researchers consider the assessment of the cognitive status of the elderly population. The cross-sectional nature of the study design, however, limits making firm conclusions on the link between geriatric depression and risk variables.

## Data availability statement

The original contributions presented in this study are included in the article/supplementary material, further inquiries can be directed to the corresponding author/s.

## Ethics statement

The studies involving human participants were reviewed and approved by University of Gondar Ethical Review Board. The patients/participants provided their written informed consent to participate in this study.

## Author contributions

AM, TA, YM, SK, GN, AK, JD, NB, DD, and HA participated in the inception of the idea, proposal development, data collection, analysis, and final write-up. All authors approved the final manuscript.
